# Using Moving Total Mortality Counts to Obtain Improved Estimates for the Effect of Air Pollution on Mortality

**DOI:** 10.1289/ehp.7774

**Published:** 2005-05-10

**Authors:** Steven Roberts

**Affiliations:** School of Finance and Applied Statistics, Faculty of Economics and Commerce, Australian National University, Canberra, Australia

**Keywords:** air pollution, distributed lag model, mortality, particulate matter, time series

## Abstract

In many cities of the United States, measurements of ambient particulate matter air pollution (PM) are available only once every 6 days. Time-series studies conducted in these cities that investigate the relationship between mortality and PM are restricted to using a single day’s PM as the measure of PM exposure. This is undesirable because current evidence suggests that the effects of PM on mortality are spread over multiple days. And studies have shown that using a single day’s PM as the measure of PM exposure can result in estimates that have a large negative bias. In this article, I introduce a new model for estimating the mortality effects of PM when only every-sixth-day PM data are available. This new model uses information available in the daily mortality time series to infer otherwise lost information about the effect of PM on mortality over a period of more than a single day. This new model typically offers an increase in both statistical estimation precision and accuracy compared with existing models.

Numerous time-series studies have investigated the association between daily mortality and some measure of daily ambient particulate matter air pollution (PM) (Chock et al. 2000; Cifuentes et al. 2000; Goldberg et al. 2003; Ito et al. 1995; Kelsall et al. 1997; Klemm et al. 2000; Kwon et al. 2001; Moolgavkar 2000; Ostro et al. 1999; Roemer and van Wijnen 2001; Smith et al. 2000; Stieb et al. 2002; Styer et al. 1995). These studies typically fit a generalized additive model (Hastie and Tibshirani 1990) or generalized linear model (McCullagh and Nelder 1989) to concurrent time series of daily mortality, PM, and meteorologic covariates. The fitted models are then used to quantify the effect of PM on mortality. The general consensus from these studies is that a 2- or 3-day moving average of PM better describes the relationship between PM and mortality than does a single day’s PM (Schwartz 2000). In addition, some recent studies have suggested that distributed lag models (DLMs) that allow differential PM mortality effects spread over multiple days may be preferable to single-day or multiple-day moving average PM exposure measures (Schwartz 2000; Smith et al. 2000). The reason is that DLMs do not leave to chance the question of how the mortality effects of PM are distributed over time.

Historically, in the United States most monitors that measure PM operate on an every-sixth-day collection schedule (Ito et al. 1995). This is a consequence of the U.S. Environmental Protection Agency often requiring PM concentrations to be collected only every sixth day. For most of the 108 cities contained in the National Morbidity, Mortality, and Air Pollution Study (NMMAPS) database (Peng et al. 2004), measurements of ambient PM < 10 μm in diameter (PM_10_) are available only once every sixth day. Consequently, in most large cities in the United States, time-series studies conducted to investigate the health effects of PM cannot use a moving average of PM or a DLM for PM. Instead, they must use a single day’s PM as the measure of PM exposure. An example of this is the recent 90-city NMMAPS analysis that was restricted to using either a lag 0, lag 1, or lag 2 PM concentration as the measure of PM exposure (Dominici et al. 2003).

The constraint of being able to use only a single day’s PM is problematic. Studies have shown that using a single day’s PM can result in a large underestimation of the relationship between PM and mortality (Roberts 2005; Schwartz 2000). The reason for this is that if the effects of PM on mortality last for > 1 day, a single-day PM exposure measure will detect the effect of PM only on 1 day’s mortality. Even worse, the wrong single-day PM exposure measure may be used. Several PM mortality time-series studies have demonstrated that the effects of PM on mortality last for multiple days (Schwartz 2000; Zanobetti et al. 2003). In addition, toxicologic evidence has shown that the morbidity effects of PM can persist for > 1 day (Clarke et al. 1999). It has been shown that DLMs avoid the problems of underestimation experienced by single-day PM exposure measures. For this reason, it has been suggested that DLMs should be the preferred measure of PM exposure if daily PM measurements are available (Roberts 2005; Schwartz 2000).

In this article, I introduce a model that typically improves both the accuracy and precision of the PM mortality effect estimates obtainable from time-series studies where PM measurements are available only every sixth day. This model uses the daily mortality time-series data to create a moving total mortality time series. The moving total mortality time series is then used in place of the current day’s mortality time series in the subsequent analysis. Simulation studies will show that for estimating the mortality effects of PM, this model offers a substantial decrease in estimation variance and typically a decrease in estimation bias compared with the standard method of using the current day’s mortality time series. With this new model, improved estimates of the effect of PM on mortality will be available for a large number of cities in the United States. This may in turn lead to a better understanding of the public health significance of PM exposure.

## Materials and Methods

### Materials

The data used in this article were obtained from the publicly available NMMAPS database (Peng et al. 2004). The data extracted consist of concurrent daily time series of mortality, weather, and PM for Cook County, Illinois, and Allegheny County, Pennsylvania, for 1987–2000. The Allegheny County data were subsequently truncated at the end of the year 1998 because PM measurements were unavailable from this time forward.

The mortality time-series data, aggregated at the level of county, are nonaccidental daily deaths of individuals ≥65 years of age. Deaths of nonresidents were excluded from the mortality counts. The weather time-series data are 24-hr averages of temperature and dew point temperature, computed from hourly observations. The measure of PM used was the ambient 24-hr concentration of PM_10_, measured in micrograms per cubic meter. PM_10_ is the most commonly used measure of PM in air pollution mortality time-series studies.

The Cook County PM time series of length 5,114 days had 251 days that were missing PM concentrations, and the Allegheny County PM time series of length 4,383 days had 24 days that were missing PM concentrations. The missing PM concentrations were imputed by taking the average of the previous and subsequent day’s PM concentration. If either the previous or subsequent day’s PM concentration was missing, the average was set equal to the nonmissing value. This method has previously been used to impute missing PM concentrations (Roberts 2004). The missing PM concentrations were imputed because a DLM of PM will be fit to the data, and missing values propagate by up to a factor of 5 when DLMs are used.

### Methods

In many community time-series studies on the effect of PM on mortality, an additive Poisson log-linear model is fit to the time series of observed mortality. Under this model, the daily mortality counts are modeled as independent Poisson random variables with a time-varying mean μ*_t_* on day *t* given by


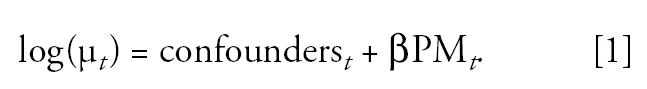


Here, confounders*_t_* represents other time-varying variables that are related to daily mortality. PM*_t_* is the time series containing the PM exposure measure, and β is the effect of this PM exposure measure on mortality. Equation 1 will be referred to as the “standard model.”

Because of data limitations, the PM exposure measure used in the standard model is typically restricted to be a single day’s PM rather than a moving average of PM or a DLM of PM. In this article, I assume that we are in such a situation; that is, only every-sixth-day PM measurements are available. As discussed above, using a single day’s PM is undesirable because it can result in estimates that have a large negative bias. And even in the unlikely event that the effect of PM on mortality is concentrated on a single day, it is possible that the wrong single-day PM exposure measure will be used. These problems would be avoided if daily PM measurements were available, making it possible for a DLM of PM to be used.

Daily mortality counts are available for cities in the NMMAPS database regardless of the sampling frequency used for PM. The model I introduce takes advantage of this fact by using information available in the daily mortality data to extract information about the effect of PM on mortality over a period of more than a single day, information otherwise unavailable with every-sixth-day PM measurements. To do this, I replace the current day’s mortality count used in the standard model with a moving total mortality count. The moving total used is a forward-moving total, meaning that the current day’s mortality count is replaced by the sum of the current day’s mortality count, the next day’s mortality, and so on, for some specified number of days. I use the term “*k* day moving total” to mean the sum of today’s and the subsequent *k* – 1 days’ mortality counts. Under this model, the *k*-day moving total mortality counts are modeled as independent Poisson random variables with a time varying mean μ*_t_*_,_*_k_* on day *t* given by


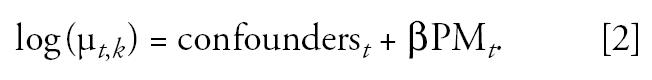


Here, confounders*_t_* has the same specification as in the standard model, and PM*_t_* is a single day’s PM, as is the case for the standard model. Simulation studies will show that the mortality effect estimates for PM obtained from Equation 2 are typically both more accurate and more precise compared with those obtained from the standard model. Equation 2 will be referred to as the “moving total model.”

A heuristic argument for why the moving total model may provide more accurate estimates of the mortality effect of PM compared with the standard model is now provided. If the mortality effect of PM lasts for more than a single day, a day of high PM will cause not only the current day’s mortality count to be elevated but also the mortality counts on subsequent days. By using a moving total mortality count, we are able to capture the increased mortality on subsequent days, information that is lost if only the current day’s mortality count is used. Obviously, if daily PM measurements are available, the best way to capture the effect of PM on mortality is through a DLM of PM. However, in the more common situation where PM measurements are available only every sixth day, using a moving total mortality count provides a “poor person’s” substitute for a DLM.

Implementing the moving total model is no harder than implementing the standard model. To fit the moving total model instead of using the current day’s mortality count (*d_t_*) as the response variable, as done in the standard model, a moving total mortality count (*d_t_*_,_*_k_*) is used instead. *d_t_*_,_*_k_* represents the *k*-day moving total mortality count for day *t*; that is, *d_t_*_,_*_k_* = d*_t_* + *d_t_*_+1_ + . . . + *d_t_*_+_*_k_*_−1_.

### Simulation Study

The simulation study compares the statistical properties of the standard model for estimating the mortality effects of PM with those of the moving total model. In the simulations, the actual weather and PM data from Cook County are used. Although the weather and PM time series are actual, the corresponding mortality time series are generated using models that describe PM mortality effects.

#### Realistic mortality generation.

To conduct the simulations, we need a way to generate realistic mortality time series. I used a method previously shown to generate realistic mortality time series (Roberts 2005), which proceeds by estimating the effects of time, temperature, dew point temperature, and day of the week on mortality using the data from Cook County. This was done by fitting the following Poisson log-linear model similar to those used in previous NMMAPS analyses (Daniels et al. 2000) to the actual Cook County mortality and meteorologic time-series data:


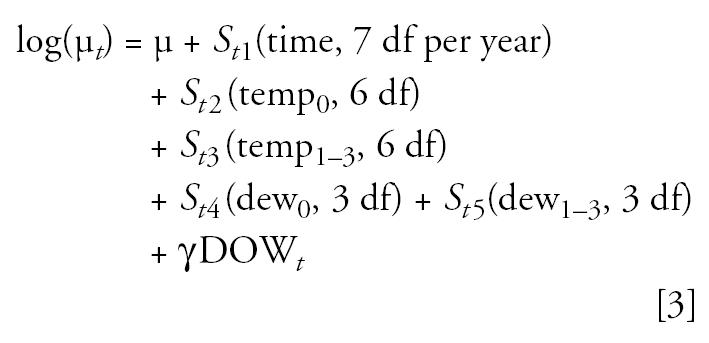


Here the subscript *t* refers to the day of the study; μ*_t_* is the mean number of deaths on day *t*; the *S_ti_*( ) are smooth functions of time, temperature, and dew point temperature with the indicated degrees of freedom (the smooth functions are represented using natural cubic splines); temp_0_ is the current day’s mean 24-hr temperature; temp_1–3_ is the average of the previous 3 days’ 24-hr mean temperatures; dew_0_ and dew_1–3_ are similarly defined for the 24-hr mean dew point temperature; DOW*_t_* is a set of indicator variables for the day of the week. All the models in this article were fit using the glm function in R (version 2.0.0; R Development Core Team 2005).

Once Equation 3 was fit, I extracted the estimated mean mortality counts, denotedμ_fit,_*_t_*. The effects of PM on mortality were explicitly specified and incorporated in the generated mortality time series. I did this by generating mortality time series that were Poisson distributed with mean ψ*_t_* on day *t* given by


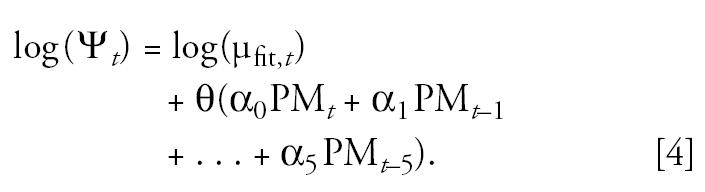


Here PM*_t_*_-_*_i_* is the time series of lag *i* PM concentrations; θ is the total mortality effect of a unit increase in PM over time (1,000θ is approximately the percentage increase in mean daily mortality for each 10-μg/m^3^ increment in PM), and θα*_i_* is the mortality effect of a unit increase in PM at lag *i*. Equation 4 assumes the mortality effects of PM can last for a maximum of 6 days.

Mortality time series were generated using various specifications for the “true” effect of PM on mortality in Equation 4. Because previous studies have shown that PM lags of more than a few days have little correlation with daily mortality (Schwartz 2000), the specifications used span a suite of plausible lag structures for the effect of PM on mortality: no effect, PM has no effect on mortality; single-day effect, the effect of PM on mortality is concentrated on a single day [the single days considered were the current day’s PM (lag 0), the previous day’s PM (lag 1), or the 2 day’s previous PM (lag 2)]; moving average effect, the effect of PM on mortality depends on a moving average of PM [the moving averages considered were the average of the current and previous day’s PM (lag 0–1), and the average of the current and previous 2 days’ PM (lag 0–2)]; distributed lag effect, differential effects of PM on mortality over time were allowed. The distributed lag effects considered were as follows:


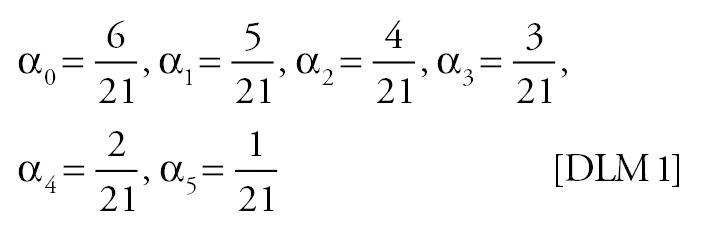



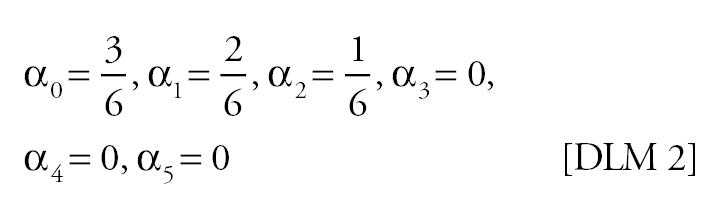



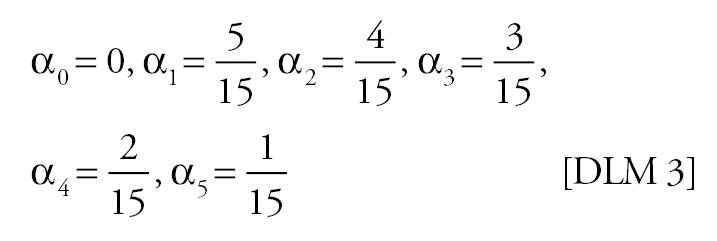



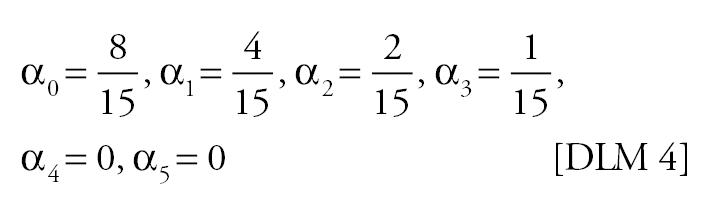


For the moving average and distributed lag effects, five θ values corresponding to 0.25, 0.5, 1, 2, and 4% increases in mortality for each 10-μg/m^3^ increment in PM were used. For the single-day effects, four θ values corresponding to 0.25, 0.5, 1, and 2% increases in mortality for each 10-μg/m^3^ increment in PM were used. These values of θ span a plausible range for the total effect of PM on mortality.

#### Fitting models to generated mortality.

For each specification of the “true” effect of PM on mortality and θ combination 400 mortality time series were generated using Equation 4. Because we are interested in the situation where PM measurements are available only every sixth day, after the mortality time series were generated, I extracted every sixth PM measurement from the PM time series of length 5,114 days that was used to generate mortality. These 852 every-sixth-day PM measurements, assumed to be the only PM measurements available, were then used in both the standard model and moving total model to estimate the effect of PM on mortality (θ). The confounders*_t_* term in both the standard and moving total models had the same specification as the confounder adjustment used in the mortality generating Equation 4. It is important to remember that in the NMMAPS database daily measurements are available for mortality, temperature, and dew point temperature irrespective of the sampling frequency used for PM.

The standard model was fit to each generated mortality time series using in turn the current day’s PM (standard model – lag 0), the previous day’s PM (standard model – lag 1), and the 2 day’s previous PM (standard model – lag 2) as the PM exposure measure (PM*_t_* in Equation 1). The moving total model was fit to each generated mortality time series using the current day’s PM as the PM exposure measure (PM*_t_* in Equation 2) and 2-, 3-, 4-, and 5-day moving total mortality counts (*k* = 2, 3, 4, 5 in Equation 2). Moving total mortality counts with *k* > 5 were not considered because the current evidence suggests that mortality counts more than a few days forward have little association with the current day’s PM concentration (Schwartz 2000). The standard and moving total models that are being fit to the generated mortality time series are identical except for the specification of the mortality response variable: For the standard models, a single day’s mortality count is used, whereas for the moving total models a moving total mortality count is used. This means that for both the standard and moving total models, the same every-sixth-day PM time series is used.

## Results

Tables 1 and 2 contain the results of the simulations. Table 1 contains the results for mortality generated using the no effect, single-day effect, and moving average effect specifications for the “true” effect of PM on mortality. Table 2 contains the results for the distributed lag effect specifications for the “true” effect of PM on mortality. These tables contain the standard deviation and bias of the estimates of the effect of PM on mortality (θ) obtained from the three forms of the standard model and the moving total models with *k* = 2, 3, and 4. A moving total model with *k* = 5 was also investigated, but the results for this model were not reported because it performed poorly compared with the moving total models with *k* = 2, 3, and 4. The reason for this is discussed further below.

Tables 1 and 2 show that the moving total models always offer a substantial reduction in estimation variance compared with the standard models. The reduction in estimation variance increases as the number of days used in the moving total mortality count (*k*) increases. The reason for this is that as the number of days used in the moving total mortality count increases, the estimates for the effect of PM on mortality are based on successively more data. For a given model, the standard deviation remains constant across the simulations because the amount of data used remains constant. Tables 1 and 2 also show that the estimation bias is typically smaller for the moving total models than for the standard models. The smaller bias for the moving total models is a consequence of the moving total mortality counts allowing the moving total models to capture the effect of PM on more than a single day’s mortality. This is something that is not possible with the standard models. For a given model, the bias increases as the total PM effect (θ) increases because the absolute amount of information lost by not observing the daily PM time series increases as θ increases. These results show that the moving total model offers a way to estimate the effect of PM on mortality that is both more precise (smaller variance) and typically more accurate (smaller bias) than the standard model.

The results reported in Tables 1 and 2 suggest that a moving total model with *k* = 2 or *k* = 3 would be preferred to moving total models with *k* ≥4. The reason for this is that *k* = 2 or *k* = 3 offers a better compromise between bias and variance. That is, the increased variance of using a moving total model with *k* = 2 or *k* = 3, as opposed to *k* ≥4, is more than offset by a decrease in bias. This is supported by the fact that in the simulations the mean squared error (for brevity, values are not reported here) for the moving total models with *k* = 2 or *k* = 3 was typically smaller than the mean squared error for the moving total models with *k* = 4 or *k* = 5. The reason for the poorer performance of the moving total models with *k* ≥4 was that in the simulations, because of evidence from previous studies (Schwartz 2000), the effect of PM on mortality was mainly concentrated at lags of up to 2 days. This meant that the last 1 or 2 days of mortality included in the moving total mortality counts when *k* = 4 or *k* = 5, respectively, were typically not associated with the measure of PM used in the model. This resulted in a dampening of the estimated effect of PM on mortality for the moving total models with *k* = 4 or 5, and hence an increase in estimation bias compared with the moving total models with *k* = 2 or *k* = 3.

### Application.

In this section I compare the results of applying the standard and moving total models to the actual Cook County and Allegheny County mortality time-series data. To do this, I first fit a DLM of PM to the mortality, meteorologic, and PM time-series data from both counties described in “Materials and Methods.” The DLM of PM contained PM concentrations lagged for 5 days, and the confounder adjustments used in this model were the same as those used in Equation 3. The effect of PM on mortality obtained from the DLM of PM was then used as a basis for judging the performance of the standard and moving total models. The rationale is that, in the ideal situation where daily PM data are available, a DLM of PM should be the method of choice for estimating the effect of PM on mortality (Roberts 2005; Schwartz 2000; Smith et al. 2000). Hence, in the situation where only every-sixth-day PM data are available, it is desirable that the method used to estimate the effect of PM on mortality return an estimate as close as possible to that obtainable in the ideal situation of daily PM data.

After fitting the DLM of PM, an every-sixth-day PM time series was obtained by extracting every-sixth-day PM concentration from the daily PM time series. With the every-sixth-day PM time series, I then estimated the effect of PM on mortality using both the standard and moving total models. The confounder adjustments used in the standard and moving total models were the same as those used in Equation 3. The estimates obtained from the standard and moving total models were then compared with the basis estimates obtained from the DLM of PM.

Table 3 contains the estimates obtained from fitting the DLM of PM, the standard models, and the moving total models to the data from both Cook County and Allegheny County. Using the estimates obtained from the DLM of PM as the basis for comparison, we can see that the moving total model with *k* = 2 provides the “best” estimates for the effect of PM on mortality. In both counties, this model produces an estimate that is closer to the basis value than the estimates obtained from the standard models. In addition, the estimate obtained from the moving total model with *k* = 2 has smaller variance than the estimates obtained from the standard models. These results reinforce the conclusions from the simulations that the moving total model offers a way to estimate the effect of PM on mortality that is both more precise and more accurate than the standard model. These results also show that the moving total model may provide a more robust estimate of the effect of PM on mortality than that obtained from the standard model. This is illustrated by the moving total models with *k* = 2, 3, and 4, avoiding the relatively poor estimates obtained from the standard model – lag 2 in Cook County and standard model – lag 1 in Allegheny County.

It is important to note the substantially smaller estimates obtained from the moving total models of Cook County data with *k* = 3 and *k* = 4 compared with that obtained with *k* = 2. The reason for this is that the large negative effect of PM on mortality observed for this data at a lag of 2 days (see standard model – lag 2) is incorporated into the estimates obtained from the moving total models with *k* = 3 and *k* = 4 but not the moving total model with *k* = 2.

## Discussion

PM air pollution is an important determinant of community health, and numerous time-series studies in the United States have investigated the association between PM and mortality (Crosignani et al. 2002; Health Effects Institute 2001). One major limitation of these studies is that in most large cities PM measurements are available only every sixth day. Time-series studies conducted in these cities cannot investigate how the effects of PM on mortality are distributed over time; instead, they are forced to examine the mortality effects of PM on a single day only. However, because the current evidence suggests that the mortality effects of PM are spread over multiple days, examining the effect of PM on a single day results in important information about the effect of PM on mortality being lost and estimates that have a large negative bias (Roberts 2005; Schwartz 2000). The moving total model introduced in this article uses information available in the daily mortality time-series data to infer some of this lost information.

The results presented here show that, for estimating the total effect of PM on mortality, the moving total model produced estimates that were substantially more precise (smaller variance) compared with those obtained from the standard model. In addition, the moving total model typically produced estimates that were more accurate (smaller bias) compared with those obtained from the standard model. These results indicate that the moving total model should be used in future air pollution mortality time-series studies where only every-sixth-day PM measurements are available.

In conclusion, because in most of the largest cities in the United States PM measurements are available only every sixth day, the moving total model has the potential, in a large number of locations, to provide improved estimates of the effect of PM on mortality that have both smaller variance and smaller bias than the estimates that are currently obtainable using existing models. This means that in multicity studies on the health effects of PM, improved estimates could be obtained for the city-specific estimates and hence for the pooled regional and national effect estimates. These improved estimates would allow researchers to better understand the health effects of PM exposure and in turn allow more informed decisions about the public health significance of PM exposure. For these reasons and the ease at which the moving total model can be implemented, I believe that the moving total model is an important contribution to the current air pollution mortality time-series methodology.

## Figures and Tables

**Table 1:** Standard deviation and bias for the estimates of the total PM effect (θ) obtained from the standard models and the moving total models.

	Model fit to generated mortality					
	Standard			Moving total		
Truth	Lag 0	Lag 1	Lag 2	*k* = 2	*k* = 3	*k* = 4
0.00^a^	0.26^b^ (−0.01)^c^	0.26 (−0.02)	0.28 (−0.01)	0.19 (0.08)	0.15 (0.11)	0.13 (0.07)
Lag 0^d^						
0.25	0.27 (0.01)	0.26 (−0.21)	0.29 (−0.24)	0.18 (0.01)	0.15 (−0.02)	0.13 (−0.08)
0.50	0.25 (0.01)	0.28 (−0.38)	0.29 (−0.49)	0.18 (−0.07)	0.15 (−0.14)	0.13 (−0.24)
1.00	0.26 (0.01)	0.28 (−0.78)	0.27 (−1.00)	0.18 (−0.25)	0.15 (−0.41)	0.13 (−0.56)
2.00	0.26 (−0.02)	0.27 (−1.57)	0.29 (−1.97)	0.18 (−0.60)	0.14 (−0.96)	0.13 (−1.22)
Lag 1						
0.25	0.27 (−0.17)	0.27 (−0.01)	0.29 (−0.19)	0.18 (0.00)	0.15 (0.00)	0.13 (−0.07)
0.50	0.26 (−0.37)	0.26 (0.00)	0.30 (−0.35)	0.19 (−0.11)	0.15 (−0.14)	0.13 (−0.22)
1.00	0.27 (−0.79)	0.27 (−0.01)	0.29 (−0.75)	0.18 (−0.33)	0.15 (−0.39)	0.13 (−0.52)
2.00	0.26 (−1.55)	0.26 (0.02)	0.28 (−1.44)	0.19 (−0.75)	0.15 (−0.89)	0.13 (−1.10)
Lag 2						
0.25	0.28 (−0.26)	0.27 (−0.20)	0.27 (0.00)	0.18 (−0.14)	0.15 (−0.05)	0.13 (−0.08)
0.50	0.27 (−0.49)	0.26 (−0.40)	0.28 (−0.03)	0.19 (−0.35)	0.14 (−0.19)	0.12 (−0.22)
1.00	0.25 (−0.95)	0.26 (−0.73)	0.29 (0.00)	0.19 (−0.78)	0.16 (−0.47)	0.14 (−0.52)
2.00	0.26 (−1.98)	0.26 (−1.50)	0.28 (0.00)	0.19 (−1.69)	0.16 (−1.08)	0.13 (−1.14)
Lag 0–1						
0.25	0.26 (−0.16)	0.25 (−0.18)	0.26 (−0.19)	0.18 (−0.08)	0.15 (−0.06)	0.12 (−0.09)
0.50	0.25 (−0.33)	0.28 (−0.32)	0.30 (−0.36)	0.18 (−0.24)	0.14 (−0.22)	0.13 (−0.26)
1.00	0.26 (−0.65)	0.28 (−0.66)	0.28 (−0.72)	0.18 (−0.55)	0.15 (−0.53)	0.14 (−0.59)
2.00	0.27 (−1.31)	0.29 (−1.34)	0.28 (−1.44)	0.19 (−1.19)	0.15 (−1.19)	0.13 (−1.27)
4.00	0.27 (−2.59)	0.25 (−2.63)	0.27 (−2.88)	0.18 (−2.46)	0.14 (−2.49)	0.13 (−2.62)
Lag 0–2						
0.25	0.27 (−0.10)	0.27 (−0.14)	0.29 (−0.18)	0.18 (−0.01)	0.15 (0.00)	0.13 (−0.07)
0.50	0.28 (−0.21)	0.26 (−0.25)	0.28 (−0.38)	0.18 (−0.14)	0.15 (−0.16)	0.13 (−0.24)
1.00	0.26 (−0.43)	0.25 (−0.52)	0.28 (−0.73)	0.18 (−0.37)	0.15 (−0.42)	0.13 (−0.55)
2.00	0.27 (−0.85)	0.26 (−1.03)	0.27 (−1.46)	0.20 (−0.83)	0.16 (−0.96)	0.14 (−1.17)
4.00	0.26 (−1.68)	0.26 (−2.06)	0.30 (−2.93)	0.19 (−1.73)	0.15 (−2.01)	0.14 (−2.40)

Truth is the specification of the “true” effect of PM on mortality and 1,000 times the θ value that were used to generate mortality.

a 1,000 times the total effect of PM on mortality (θ) that was used to generate mortality.

b 1,000 times the standard deviation for the estimate of the total effect of PM on mortality (θ).

c 1,000 times the bias for the estimate of the total effect of PM on mortality (θ).

d The specification of the “true” effect of PM on mortality that was used to generate mortality.

**Table 2:** Standard deviation and bias for the estimates of the total PM effect (θ) obtained from the standard models and the moving total models.

	Model fit to generated mortality					
	Standard			Moving total		
Truth	Lag 0	Lag 1	Lag 2	*k* = 2	*k* = 3	*k* = 4
DLM 1^a^						
0.25^b^	0.27^c^ (−0.16)^d^	0.26 (−0.11)	0.28 (−0.16)	0.19 (−0.06)	0.16 (−0.03)	0.13 (−0.08)
0.50	0.27 (−0.31)	0.27 (−0.27)	0.28 (−0.27)	0.19 (−0.20)	0.16 (−0.17)	0.13 (−0.24)
1.00	0.26 (−0.61)	0.28 (−0.52)	0.29 (−0.57)	0.18 (−0.47)	0.15 (−0.44)	0.13 (−0.55)
2.00	0.25 (−1.15)	0.27 (−0.99)	0.27 (−1.12)	0.18 (−0.99)	0.14 (−0.97)	0.12 (−1.15)
4.00	0.26 (−2.33)	0.26 (−2.03)	0.28 (−2.29)	0.18 (−2.10)	0.15 (−2.07)	0.13 (−2.39)
DLM 2						
0.25	0.26 (−0.11)	0.27 (−0.08)	0.27 (−0.22)	0.20 (0.00)	0.15 (−0.01)	0.13 (−0.07)
0.50	0.27 (−0.22)	0.27 (−0.20)	0.27 (−0.42)	0.19 (−0.12)	0.16 (−0.15)	0.14 (−0.24)
1.00	0.27 (−0.40)	0.26 (−0.39)	0.28 (−0.85)	0.19 (−0.29)	0.16 (−0.41)	0.14 (−0.54)
2.00	0.27 (−0.79)	0.27 (−0.76)	0.29 (−1.70)	0.18 (−0.68)	0.15 (−0.92)	0.13 (−1.15)
4.00	0.26 (−1.56)	0.28 (−1.56)	0.28 (−3.39)	0.19 (−1.43)	0.15 (−1.96)	0.13 (−2.40)
DLM 3						
0.25	0.25 (−0.22)	0.26 (−0.16)	0.26 (−0.14)	0.18 (−0.11)	0.15 (−0.07)	0.13 (−0.10)
0.50	0.27 (−0.46)	0.27 (−0.31)	0.29 (−0.35)	0.19 (−0.30)	0.16 (−0.24)	0.14 (−0.26)
1.00	0.28 (−0.92)	0.27 (−0.62)	0.27 (−0.63)	0.19 (−0.66)	0.16 (−0.58)	0.13 (−0.61)
2.00	0.27 (−1.81)	0.27 (−1.22)	0.27 (−1.23)	0.19 (−1.42)	0.15 (−1.27)	0.13 (−1.29)
4.00	0.26 (−3.64)	0.25 (−2.48)	0.27 (−2.46)	0.19 (−2.94)	0.15 (−2.67)	0.13 (−2.66)
DLM 4						
0.25	0.26 (−0.13)	0.26 (−0.15)	0.29 (−0.22)	0.19 (−0.04)	0.15 (−0.04)	0.13 (−0.08)
0.50	0.25 (−0.21)	0.26 (−0.29)	0.27 (−0.41)	0.18 (−0.16)	0.15 (−0.17)	0.14 (−0.24)
1.00	0.27 (−0.40)	0.26 (−0.59)	0.29 (−0.77)	0.20 (−0.38)	0.16 (−0.44)	0.14 (−0.55)
2.00	0.28 (−0.80)	0.26 (−1.16)	0.27 (−1.53)	0.19 (−0.86)	0.15 (−1.00)	0.13 (−1.18)
4.00	0.26 (−1.61)	0.27 (−2.32)	0.29 (−3.11)	0.18 (−1.81)	0.16 (−2.11)	0.13 (−2.42)

Truth is the specification of the “true” effect of PM on mortality and 1,000 times the θ value that were used to generate mortality.

a The specification of the “true” effect of PM on mortality that was used to generate mortality.

b 1,000 times the total effect of PM on mortality (θ) that was used to generate mortality.

c 1,000 times the standard deviation for the estimate of the total effect of PM on mortality (θ).

d 1,000 times the bias for the estimate of the total effect of PM on mortality (θ).

**Table 3:** Results of fitting both the standard and moving total models to the actual data from Cook County, Illinois, and Allegheny County, Pennsylvania.

	Model fit to mortality						
	Standard			Moving total			
County	Lag 0	Lag 1	Lag 2	*k* = 2	*k* = 3	*k* = 4	Baseline
Cook County	0.127^a^ (0.264)^b^	−0.042 (0.249)	−0.441 (0.246)	0.150 (0.187)	−0.047 (0.153)	0.009 (0.133)	0.462 (0.212)
Allegheny County	0.693 (0.437)	0.356 (0.423)	0.524 (0.415)	0.633 (0.310)	0.542 (0.255)	0.528 (0.221)	0.598 (0.351)

Baseline is the baseline estimate of the total effect of PM on mortality obtained from the DLM of PM fit to the daily data.

a 1,000 times the estimated effect of PM on mortality.

b 1,000 times the standard deviation of the estimated effect of PM on mortality.
